# Effect of Pulsed Current on the Tensile Deformation Behavior and Microstructure Evolution of AZ80 Magnesium Alloy

**DOI:** 10.3390/ma13214840

**Published:** 2020-10-29

**Authors:** Hong Xu, You Zhou, Yu-Jie Zou, Meng Liu, Zhi-Peng Guo, Si-Yu Ren, Rong-Hui Yan, Xiu-Ming Cheng

**Affiliations:** Key Laboratory of Automotive Materials of Ministry of Education & School of Materials Science and Engineering, Nanling Campus, Jilin University, No. 5988 Renmin Street, Changchun 130025, China; xh@jlu.edu.cn (H.X.); zhouyou17@mails.jlu.edu.cn (Y.Z.); zouyj18@mails.jlu.edu.cn (Y.-J.Z.); liumeng18@mails.jlu.edu.cn (M.L.); guozp18@mails.jlu.edu.cn (Z.-P.G.); syren19@mails.jlu.edu.cn (S.-Y.R.); yanrh19@mails.jlu.edu.cn (R.-H.Y.)

**Keywords:** pulsed current, deformation behavior, microstructure evolution, magnesium alloys

## Abstract

In this work, the tensile deformation behavior of an as-extruded AZ80 magnesium alloy under pulsed current (PC) was investigated based on microstructure observations. We found that compared with the tensile tests at room temperature (RT) and given temperature (GT), the flow stress is reduced due to both thermal and athermal effects of pulsed current. A quasi-in-situ electron backscatter diffraction (EBSD) analysis reveals that at the same strain, the geometrically necessary dislocation (GND) density of the RT sample is the highest, followed by the GT sample and the PC sample. This proves that the athermal effect can promote the annihilation of dislocations and slow down dislocation pileup, which reduces the flow stress. In addition, the twinning behavior under different deformation conditions was studied; the twins are {10−12} tension twins, which are activated with the assistance of local stress. We found that the twin fraction in the PC sample is lower than that in the RT and GT samples, due to the least accumulation of GNDs at grain boundaries, which decreases the nucleation of {10−12} tension twins.

## 1. Introduction

Recently, reducing the weight of vehicles and improving fuel efficiency have become important issues in the automotive industry [1,2]. Due to the low density and high specific strength of magnesium alloys [3,4], the global demand for magnesium alloys is increasing. However, the commercial application of magnesium alloys is still restricted because of their poor formability compared with conventional aluminum alloys, which is attributed to the limited number of active slip systems of the hexagonal close-packed crystal structure at room temperature and the strong basal texture [5]. In order to overcome these drawbacks, hot forming and incremental forming have frequently been considered [6,7]. Nevertheless, the above forming methods still have drawbacks, such as the adhesion between the die and alloys, high processing costs, and inefficient processing [8]. In addition, due to the relatively high friction coefficient, low wear resistance, and a rapid corrosion rate, the commercial application of magnesium alloys in mechanical and biological engineering is also severely restricted. Creating a surface layer on magnesium alloys is usually considered an effective solution. Kaczmarek et al. creatively proposed that depositing Al/AlC/α-C:H coatings on the surface of the AZ31 magnesium alloy can improve wear resistance and achieve a smooth transition of mechanical properties between the α-C:H coating and the AZ31 substrate [9]. Liu et al. suggested a combined coating based on atomic layer deposition, which improved the corrosion resistance of the AZ31 magnesium alloy in physiological saline [10].

It was reported that applying electric current through metals and alloys can enhance plastic deformation by reducing the flow stress and increasing the ductility [11]; this is often referred to as electroplastic [12]. The discovery of such a phenomenon leads to a new method for metallic material forming, which is known as electrically-assisted manufacturing. Various studies on the mechanism of electroplastic have suggested that the mechanical behavior under an electric current may be described satisfactorily in terms of (1) the thermal effect due to Joule heating and (2) the athermal effect due to electric current, such as the electron wind [13,14] and magnetic field [15,16].

Magargee et al. showed that the thermal-mechanical constitutive models can effectively predict the measured mechanical behavior of commercially pure titanium during a tensile test with a continuous DC current, without the need for electroplastic theory [17]. Li et al. proposed that Joule heating caused by pulsed current is an important reason for the high forming quality of the AZ31 alloy in the free-bulging tests [18]. On the contrary, it was also reported that the deformation behavior under electric current can be explained by the athermal effect of electroplastic. Kuang et al. [19] showed that pulsed current could improve the rolling ability of the AZ31 alloy, which probably originated in the improved strain compatibility due to enhanced prismatic slip activity by the athermal effect. Kim et al. observed the annihilation of dislocations by applying pulsed current during tensile tests of a 5052 aluminum alloy with a distinct effect from Joule heating [20]. In addition, it was also suggested that the athermal effect of pulsed current can accelerate dissolution of the Mg_17_Al_12_ phase in the AZ91 alloy, resulting in the noticeably improved ductility [21].

Even though quite a few studies have been conducted on the effect of pulsed current on deformation, Joule heating caused by high current density often concealed the athermal effect. Moreover, the electroplastic at low temperatures has been less investigated. In view of the above research status, a low density and high frequency pulsed current is designed for the first time to assist tensile deformation in this work. The original intention of this design is to weaken the thermal effect that is caused by Joule heating and enhance the athermal effect. The purpose of this work is to investigate the effect of pulsed current on the tensile deformation behavior of the AZ series of magnesium alloys. The tensile tests under room temperature, pulsed current, and given temperatures are carried out. By comparing the tensile properties under room temperature (RT), pulsed current (PC), and given temperature (GT) conditions, the thermal and athermal effects are distinguished. In addition, due to the low deformation temperature, the surface oxidation degree of tensile test samples is light. The deformation behavior associated with thermal and athermal effects was discussed based on the continuous and dynamic microstructure evolution analysis by quasi-in-situ electron backscatter diffraction (EBSD).

## 2. Materials and Methods

**Figure 1:**
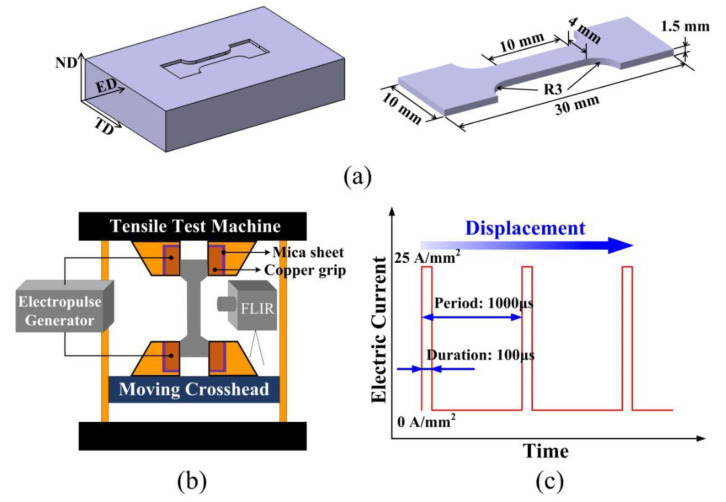
Schematics of (**a**) samples used for tensile tests; (**b**) setup for pulsed current tensile tests; and (**c**) pulsed current applied to samples during tensile test.

A commercial extruded AZ80 (Mg-8.0Al-0.5Zn-0.3Mn, wt%) magnesium alloy sheet with a 350 mm width and 13.5 mm thickness was used in this work. It was indirectly extruded at 330 °C, with an extrusion speed of 0.5 mm/s and an extrusion ratio of 26. As shown in Figure 1a, tensile samples were cut from the center of as-extruded sheets surface using electrical discharge machining (EDM), with the tensile axis aligned along the extrusion direction (ED). Uniaxial tensile tests were conducted at a constant speed of 0.6 mm/min (corresponding to a nominal strain rate of 0.001 s^−1^). As described in Figure 1b, the tensile test machine was modified to make electric current pass only through the sample by inserting a mica sheet between the copper jigs and the sensor. The pulsed current was generated by a 4000 FN DC power supply (CHANT, Zibo, China) and periodically applied to the tensile sample with a duration of 100 μs and a period of 1000 μs (Figure 1c).

According to the initial cross section area of the sample, the average current density of ≈2.5 A/mm^2^ is nearly a constant before necking. The temperature of samples during the pulsed current tensile test was measured by the E95 infrared thermal imaging camera (FLIR, Stockholm, Sweden). Black paint was sprayed on one side of the sample facing the FLIR camera to stabilize the emissivity of the sample. It should be noted that the temperature measured using the FLIR camera system was calibrated using a K-type thermocouple (Songdao, Shanghai, China). To investigate the athermal effect of pulsed current, a tensile test at a given temperature (GT) was also conducted using a tensile test machine (SHIMAZU AGS-X, Kyoto, Japan). It should be noted that six samples in each tensile deformation group (i.e., RT, PC, and GT conditions) were tested, and engineering stress–strain curves with good repeatability were produced.

To analyze the microstructure evolution of samples during the RT, PC, and GT tensile tests, quasi-in-situ EBSD measurements were conducted via tracking of a selected area at different strains (0, 0.04, and 0.08), using AZtec and channel 5.0 data acquisition software. The advantage of quasi-in-situ EBSD is that its observation of the microstructure is continuous and dynamic. However, it is also required that the sample has strong oxidation resistance to ensure a good EBSD scanning quality. Research suggested that the oxidation behaviors may be related to the aluminum content for magnesium alloys, which the oxide changes from bulk to thin film with the increment of Al content [22]. Therefore, the AZ80 magnesium alloy with high Al content was selected in the present work. As shown in Table 1, samples are denoted based on different strains under RT, PC, and GT conditions.

In the EBSD analysis, grains with misorientation angles larger than 7.5° and less than 1° were regarded as deformed grains and recrystallized grains, respectively [23]. The substructured grains were defined with misorientation angles ranging from 1° to 7.5° [24].

## 3. Results and Discussion

### 3.1. Deformation Behavior

In this section, the effect of pulsed current on the deformation behavior of the AZ80 magnesium alloy, especially the reduction of flow stress in the homogeneous deformation stage, is studied. By comparing the tensile properties under RT, PC, and GT conditions, thermal and athermal effects are discussed respectively.

**Figure 2:**
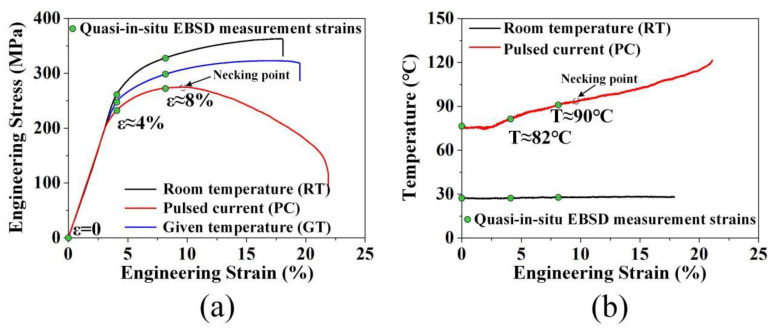
(**a**) Engineering stress–strain curves of tensile tests at room temperature (RT, black line), pulsed current (PC, red line), and given temperature (GT, blue line); (**b**) The temperature of samples measured during tensile tests at room temperature (RT, black line) and pulsed current (PC, red line).

The typical tensile engineering stress–strain curves under RT, PC, and GT conditions are shown in Figure 2a, with the corresponding tensile properties listed in Table 2. Compared with samples deformed at RT, the PC sample shows lower yield strength (273 MPa→215 MPa) and ultimate tensile strength (363 MPa→273 MPa). In addition, the flow stress under pulsed current at the plastic deformation stage is reduced. The present work focuses on the homogeneous deformation stage (0 ≤ ε ≤ 0.08) and studies the mechanism of a relative reduction of flow stress caused by pulsed current.

Figure 2b shows the measured temperature during PC tensile test. Due to the input of pulsed current, temperature of the sample rises rapidly to about 82 °C at the strain of 0.04 and gradually reaches to about 90 °C at the strain of 0.08. In order to distinguish the Joule heating effect (thermal effect) and the athermal effect on tensile properties, given temperature (GT) tensile tests were conducted for comparison at 100 °C, which is higher than the measured temperature in the strain range of 0–0.08. The flow stress of the GT sample is lower than that at RT but higher than that at PC during deformation, indicating that the relative reduction of flow stress in the PC sample is caused by both thermal and athermal effects.

With regard to the effect of pulsed current on flow stress, it is generally considered that the Joule heating effect can soften the alloy during deformation [20,21]. In the strain range of 0–0.08, the temperature of the PC sample (77–90 °C) was much lower than the annealing (200 °C [25]) and dynamic recrystallization (DRX) temperature (300 °C [26]) of the AZ80 magnesium alloy. Therefore, it is assumed that recovery occurs in the AZ80 magnesium alloy under pulsed current in the present work.

### 3.2. Microstructure Evolution under Different Deformation Conditions

In this section, we mainly establish the micromechanics of pulsed current to reduce the flow stress of the AZ80 magnesium alloy. By comparing dislocation multiplication under RT, PC, and GT conditions, the thermal and athermal effects on flow stress and microstructure evolution are discussed.

**Figure 3:**
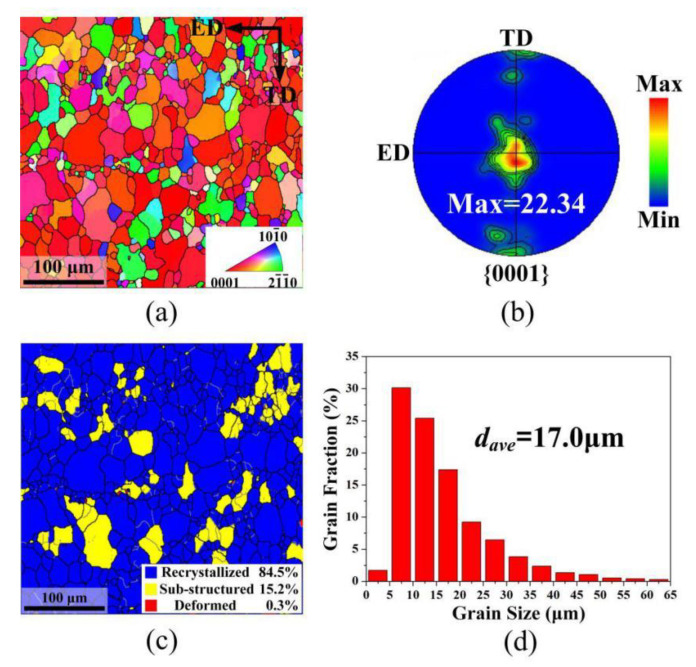
Microstructure and texture of as-extruded AZ80 alloy: (**a**) electron backscatter diffraction (EBSD)-inverse pole figure (IPF) map; (**b**) {0001} pole figure; (**c**) grain type distribution maps (blue, recrystallized; yellow, substructured; red, deformed); and (**d**) grain size distribution.

Figure 3 shows the EBSD map, {0001} pole figure, grain type, and grain size distribution maps of the as-extruded AZ80 magnesium alloy. As shown in Figure 3a,b, the as-extruded alloy shows a strong basal texture with a maximum intensity of 22.34. As indicated in Figure 3c, the as-extruded alloy mainly consists of recrystallized grains (area fraction of 84.5%) with an average size of ≈17 μm.

**Figure 4:**
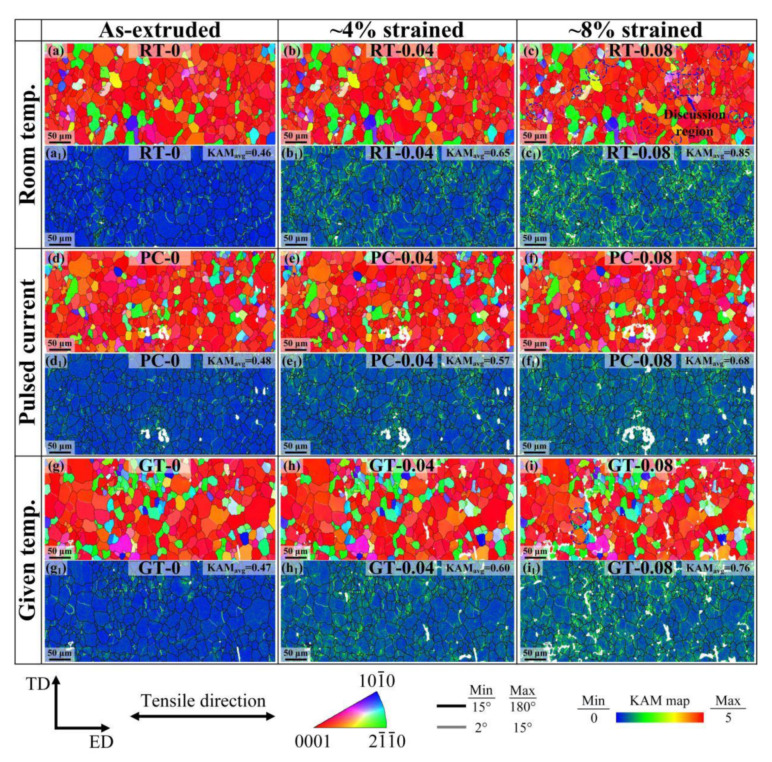
EBSD and kernel average misorientation (KAM) maps of the samples deformed under different conditions (average KAM are given in upper-right corners).EBSD maps showing the quasi-in-situ observation in (**a**–**c**) RT-0, RT-0.04 and RT-0.08, (**d**–**f**) PC-0, PC-0.04 and PC-0.08, (**g**–**i**) GT-0, GT-0.04 and GT-0.08; (**a_1_**–**i_1_)** KAM maps corresponding to the EBSD map of (**a**–**i**) respectively.

To further study changes in the flow stress of the AZ80 magnesium alloy under different tensile conditions, the microstructure of the as-extruded alloy under RT, PC, and GT conditions at various strains were studied using the quasi-in-situ EBSD method. It is well known that the plastic deformation of metals is dominated by dislocation movements. With the increment of strains, the dislocation multiplication and pileup lead to the work hardening and the increment of flow stress. As shown in Figure 4a,d,g, the microstructures of RT-0, PC-0, and GT-0 consist mainly of equiaxed grains with the average sizes of 17.3 μm, 16.9 μm, and 17.0 μm, respectively. In addition, all of them have a typical basal texture. In Figure 4a–i, the grains of RT, PC, and GT samples, respectively, were slightly elongated along tensile direction with the increment of strains, but the grain size and orientation substantially unchanged. 

Compared with the RT and GT or PC samples (Figure 2a), the flow stress decreases with the increase of deformation temperature. This is because the thermal energy activates the recovery process and annihilates the dislocations produced during tensile deformation. However, the temperature change cannot explain the difference of flow stress between the sample GT and PC. Therefore, it is assumed that the athermal effect of pulsed current reduces the flow stress by affecting dislocation multiplication. The effect of pulsed current on the evolution of dislocation density is discussed below.

**Figure 5:**
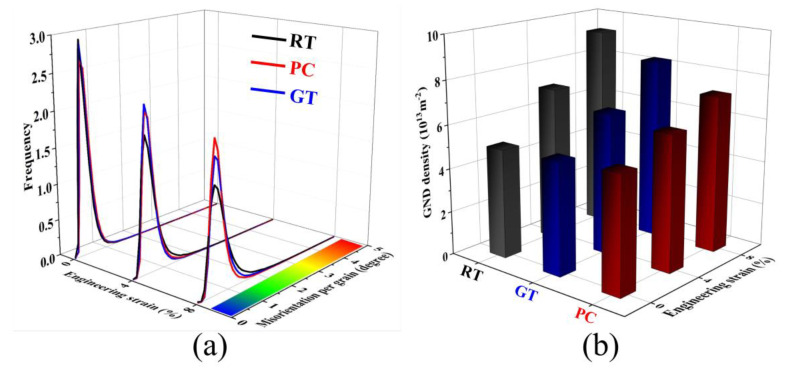
(**a**) The distribution of misorientations per grain and (**b**) geometrically necessary dislocation (GND) density.

Figure 4a_1_–i_1_ shows the kernel average misorientation (KAM) maps corresponding to Figure 4a–i. In general, the KAM has been widely accepted and adopted as an indicator of dislocation density during the plastic deformation of metallic materials [27]. Through the distribution of misorientation per grain for each area as shown in Figure 5a, the average KAM values (KAM_avg_ marked in Figure 4) were calculated. It can be seen that the KAM_avg_ of RT-0, PC-0, and GT-0 are very low, i.e., 0.46, 0.48, and 0.47, respectively. This is because the dislocations formed during hot extrusion are annihilated through DRX after the annealing treatment. The evolution of KAM_avg_ in the RT sample is shown in Figure 4a_1_–c_1_. At the strain of 0.04, the dislocations pile up, resulting in an increase of KAM_avg_ from 0.46 to 0.65. With the strain increasing to 0.08, the average KAM increases to 0.85, while in the GT sample, KAM_avg_ increases from 0.47 to 0.76. The lowest increment of KAM_avg_ is in the PC sample (from 0.48 to 0.68). Gao et al. [28] revealed that the density of geometrically necessary dislocations (GNDs) has a correlation with the KAM value, as shown in the following equation:

(1) $$\rho^{\mathrm{GND}}=\frac{2\theta }{\mu b}$$

where *θ*, *μ*, and b are the average KAM (in radian), scanning step, and Burger’s vector, respectively. In this work, *θ* is calculated by the misorientation data, *μ* is 1 μm, and b is 0.321 [29]. As shown in Figure 5b, with the same strain (0.04 and 0.08), the GND density of the RT sample is the highest, followed by the GT sample, and then the PC sample. 

Regarding the evolution of GND density, the thermal energy activates the recovery process, which promotes the elimination of the opposite sign dislocations and decreases the dislocation density. In addition, previous studies in the electroplastic effect with high Joule heating have suggested that pulsed current can promote the de-pinning of dislocations from obstacles [30], and the electron wind force can promote the dislocation mobility [31]. In theory, both thermal and athermal effects have positive effects on annihilating dislocations, which is consistent with the increment of the GND density formed locally by microscopic plastic instability and further restrained under pulsed current. 

Regarding the distribution of GND, the KAM maps show that at the strain of 0.08, GNDs are highly concentrated near grain boundaries (GBs) in the RT sample. This is because when polycrystalline metal undergoes plastic deformation, local stress is generated at GBs to ensure plastic strain compatibility between adjacent grains [32] and leads to an increase in the GND density near the GBs. After comparison, the accumulation of GNDs at the GBs is the least in the PC-0.08 sample. It indicates that the athermal effect can slow down the dislocation piling up and alleviate the concentration of local stress in GBs, which helps to avoid fatigue failure of the sample.

**Figure 6:**
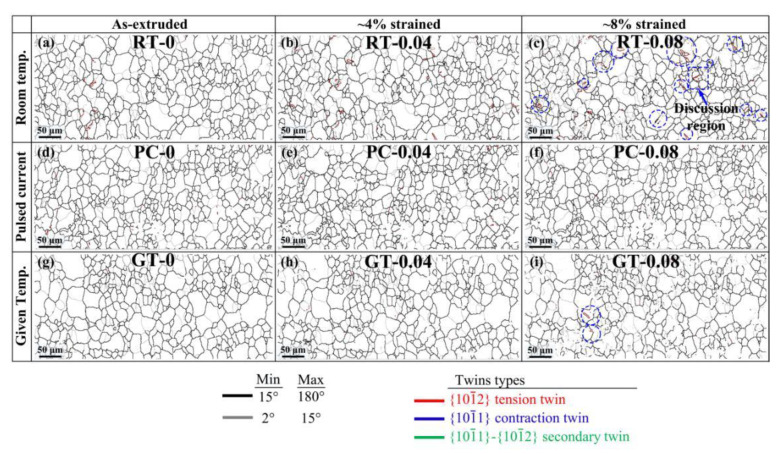
Boundary maps of (**a**–**c**) RT-0, RT-0.04 and RT-0.08; (**d**–**f**) PC-0, PC-0.04 and PC-0.08; (**g**–**i**) GT-0, GT-0.04 and GT-0.08 which correspond to each sub-map in Figure 4.

Pulsed current not only reduces the dislocation density, but it also affects the twinning behavior. In the boundary maps in Figure 6, all the twins types are {10−12} tension twins, while the {10−11} contraction twins and {10−11}-{10−12} double twins cannot be observed. This is because the {10−12} tension twins usually possess lower critical resolved shear stress (CRSS) and are susceptible to activate at the initial stage of deformation [33]. Although the twins types are the same, the twin fraction under RT, PC, and GT conditions are different. It is well known that the twin fraction of magnesium alloy increases with increasing strains [34]. The number of parent grains containing activated twins in different samples at the strain of 0.08 (blue circle in Figure 4 and Figure 6) were compared. It can be seen that in the same area, the number of activated twins in the RT-0.08 sample is the largest, followed by the GT-0.08 sample, and no twins were observed in the PC-0.08 sample. This indicates that the twins fraction and flow stress have a corresponding relationship, i.e., pulsed current exhibits an inhibiting effect on twin activation. 

**Figure 7:**
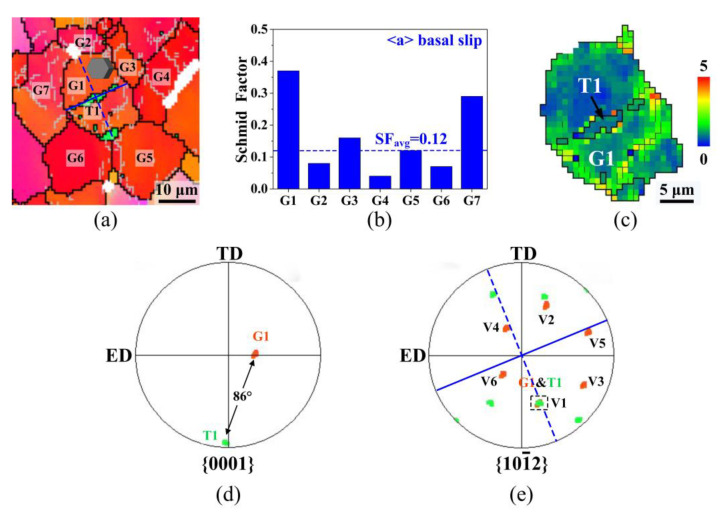
(**a**) EBSD map of the framed area by blue dotted border in Figure 4c; (**b**) Schmid factors of basal slip in G1–7; (**c**) KAM map of G1 and T1; (**d**) shows {0001} pole figure of G1 and T1; and (**e**) shows {10−12} pole figure of G1 and T1. Note that the color code is the same as that in Figure 3.

To reveal the connection between the twins fraction and flow stress, it is necessary to analyze the nucleation of twins in this work. Here, we discuss twinning behavior using an activated twin in Figure 4c as an example. Figure 7a shows the EBSD map of G1 and its surrounding grains, and it can be seen that a lamella twin T1 has formed in the parent grain G1 before the strain reached to 0.08. The orientations of G1 and T1 in {0001} and {10−12} pole figures are shown in Figure 7d,e, respectively. It can be seen that T1 exhibited a misorientation angle of ≈86° relative to G1 with a common {10−12} axis, which suggests that T1 is a {10−12} tension twin. It is known from geometrical consideration that the normal {10−12} tension twins are formed under tension in the C-axis [33]. However, the hexagonal unit cell of G1 in Figure 5a shows that the angle between the C-axis and tensile direction is ≈68°, which is the relative hard orientation for G1 to activate {10−12} tension twins. According to the previous studies [35,36], the nucleation of such anomalous {10−12} tension twins are often related to the strain compatibility that affects twinning behavior according to the local stress state. 

The activated variant twin was identified using the twinning rotation method [37] and twin trace method [38]. Basing on the twinning rotation method, the misorientation (θ) between the theoretical variants and observed twin variant were calculated and are shown in Table 3. The twin variant with the minimum misorientation angle was determined to be the activated one. According to the twin trace method, considering that the twin and its parent grain share one {10−12} twinning plane, the coincidence position of G1 and T1 on the {10−12} pole figure indicates the activated variant type, and the position of the shared plane is normal to the orientation of the twin trace as shown in Figure 7e. From Table 3 and Figure 7e, the variant type of T1 is identified as (−1102)[1−101], which has the highest Schmid factor (SF) of 0.082 among 6 variants. Considering that 0.082 is much lower than the theoretical maximum SF (0.5), it is necessary to analyze the stress state during the twin nucleation of T1. Yang et al. [39] reported that the CRSS for {10−12} tension twinning of the AZ80 magnesium alloy with similar average grain size is ≈45 MPa. According to Schmid’s law, the flow stress (≈330 MPa) of RT-0.08 is lower than the theoretical activation stress (543 MPa) of T1. Therefore, the nucleation of T1 could only occur with the assistance of local stress. It is well known that the accumulated local stress of magnesium alloys can be induced by an intensive basal slip. D. Ando et al. [40] suggested that the basal slip induces twinning in the same grain to accommodate strain compatibility with the surrounding grains. Comparing the SF of the basal slip of G1 and its surrounding grains, as shown in Figure 7b, the parent grain G1 has the highest SF (0.37) in the region, which is much higher than the SF_avg_ (0.12) of all grains in RT-3. This indicates that the basal slip in G1 is more intensive than that in other grains, which causes dislocation pileup and local stress. Figure 7c shows the KAM map of G1. Obviously, higher misorientations are distributed in G1, indicating the accumulation of high local stress here.

**Figure 8:**
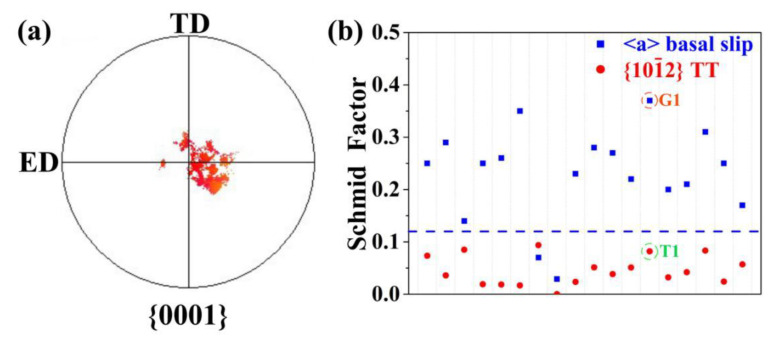
(**a**) {0001} pole figure of the parent grains containing activated {10−12} tension twins in the RT-0.08 sample; (**b**) Schmid factors of basal slip and {10−12} tension twinning in RT-0.08 sample.

The accumulated local stress makes the stress state reach the stress threshold of T1 nucleation, but it also means that the region has serious work hardening which corresponds to the higher flow stress. From the statistical analysis of twins in RT-0.08, as shown in Figure 8a,b, most of the parent grains have the basal plane orientation and a higher SF of basal slip, while the activated variants have a lower SF of {10−12} tension twinning. It shows that the nucleation mechanism of {10−12} tension twins in this work is affected by the strain compatibility. Since the area where twins activate means an accumulated local stress and serious work hardening, the highest twins fraction in the RT sample corresponds to its highest flow stress. Due to the annihilation of dislocation by thermal and athermal effects, the accumulated local stress is relieved, and the stress threshold for twin activation is not reached, which explains the inhibiting effect of twin activation by pulsed current. 

According to the above analysis, the flow stress affects the {10−12} tension twinning behavior. It should be noted that activated twins also affect the flow stress. The specific strain provided by twinning can be calculated using the following equation [33]:

(2) $$\varepsilon_{\mathrm{twin}}=f_{\mathrm{twin}}\cdot m\cdot \gamma_{\mathrm{twin}}$$

where *ε*_twin_, f_twin_, m, and *γ*_twin_ are the twinning strain, area fraction of twins, SF_avg_ of {10−12} extension twins, and the characteristic twinning shear, respectively. Taking the RT-0.08 sample as an example, when the strain is 0.08, f_twin_ is 2.1%, m is 0.069, and *γ*_twin_ is 0.129, and accordingly, *ε*_twin_ is calculated to be 0.019%. It indicates that the softening effect of {10−12} extension twinning during tensile deformation is extremely small, and the hardening effect is more obvious. As obstacles to dislocation movements, twin boundaries lead to the dislocation multiplication and enhance the work hardening effect of the alloy [41]. It is proven by the distribution of a higher misorientation at the twin boundary of T1, as shown in Figure 7c. It can be considered that for the RT sample with the largest twin fraction, the enhancement of the grain boundary strengthening by twin boundaries is the most significant, which is one of the internal reasons of the increment of flow stress. Since no twins are observed in the PC-0.08 sample, the increment of flow stress caused by twin boundaries does not exist, which is one of the internal reasons for the lower flow stress under pulsed current.

## 4. Conclusions

In this work, the effect of pulsed current on the reduction of tensile flow stress in an as-extruded AZ80 magnesium alloy was investigated using quasi-in-situ EBSD. The following conclusions can be drawn:

(1) Compared to the tensile tests at RT and GT, when pulsed current is applied to the sample during tensile deformation, the flow stress decreases, which is caused by both the thermal and athermal effects.
(2) Thermal and athermal effects annihilate the dislocations produced during tensile deformation. At the same strain, the GND density of the PC sample is the lowest, which leads to the reduction of flow stress. Meanwhile, pulsed current can slow down the dislocation piling up and can alleviate the concentration of local stress in GBs.
(3) Through annihilating dislocations and alleviating the concentration of local stress, pulsed current inhibits {10−12} tension twin activation.

Based on the experimental results in this work, we identified that low density and high frequency pulsed current is effective in formability improvement, as a result of both the athermal effect and Joule heating effect. Furthermore, our results indicate that an athermal effect can promote the recovery process of the AZ80 magnesium alloy during deformation. These results provide important insights to the development of electrically-assisted manufacturing techniques and their industrial applications.

## Figures and Tables

**Table 1:** Samples are denoted according to deformation conditions and strains.

	ε = 0	ε = 0.04	ε = 0.08
Room Temperature (RT)	RT-0	RT-0.04	RT-0.08
Pulsed Current (PC)	PC-0	PC-0.04	PC-0.08
Given Temperature (GT)	GT-0	GT-0.04	GT-0.08

**Table 2:** Tensile properties at RT, PC, and GT conditions of as-extruded AZ80 magnesium alloy in this work.

	Yield Strength (MPa)	Ultimate Strength (MPa)
Room Temperature (RT)	273.1 ± 0.7	363.6 ± 2.5
Pulsed Current (PC)	214.7 ± 0.3	272.6 ± 1.8
Given Temperature (GT)	236.7 ± 0.6	324.5 ± 2.1

**Table 3:** Misorientation (*θ*) between the theoretical twin variants and observed twin variant in G1 (activated twin variant marked in bold).

G1 (Euler Angle: 93.0°, 24.2°, 52.6°)			
Twin Variants	Twining System	Misorientation (*θ*)	Schmid Factor
**V1**	**(−1102)[1−101]**	**1.8**	**0.082**
V2	(1−102)[−1101]	86.4	0.078
V3	(−1012)[10−11]	39.5	−0.221
V4	(10−12)[−1011]	73.2	−0.181
V5	(0−112)[01−11]	72.7	−0.274
V6	(01−12)[0−111]	39.8	−0.231
